# Performance of silver, zinc, and iron nanoparticles-doped cotton filters against airborne *E. coli* to minimize bioaerosol exposure

**DOI:** 10.1007/s11869-018-0622-0

**Published:** 2018-09-15

**Authors:** Attarad Ali, Maohua Pan, Trevor B. Tilly, Muhammad Zia, Chang Yu Wu

**Affiliations:** 1 Department of Environmental Engineering Sciences, University of Florida, Gainesville, FL 32611-6450 USA; 2 Department of Biotechnology, Quaid-i-Azam University, Islamabad, 45320 Pakistan

**Keywords:** Cellulose, Nanocomposites, Aerosols, Removal efficiency, Biocidal filter

## Abstract

To overcome limitations of existing air-cleaning filters in capturing and deactivating aerosolized microorganisms, this study was embarked to evaluate novel Ag, Zn, and Fe nanoparticle-doped cotton filters (AgCt, ZnCt, FeCt), as biocidal filters for bioaerosol attenuation. To evaluate the biocidal activity of the nanocomposite filters, the survival of lab-generated *E. coli* after collection on each filter material was compared to collection on an undoped cotton control filter and in a BioSampler. Relative humidity (RH) affected the survival of bacteria on the filters, and the optimal RH was found to be 50 ± 5%. The physical removal efficiency (PRE) determined by an optical particle counter was 99.9 ± 0.7% for ZnCt, 97.4 ± 1.2% for AgCt, and 97.3 ± 0.6% for FeCt, where the control showed only 77.4 ± 6.3% for particles > 500 nm. The doped filters showed 100% viable removal efficiency (VRE). Importantly, the VRE of the nanocomposite filters after four cycles remained nearly 99% and was greater than the cotton control filter at 76.6 ± 3.2%. Adding to its benefits, the AgCt filters had a lower pressure drop than the FeCt and ZnCt filters and the cotton control. The permeability for the cotton control filter was 3.38 × 10^−11^ m^2^ while that for the AgCt filter was slightly higher (3.64 × 10^−11^ m^2^) than the other filters as well. Overall, these results suggest that nanocomposite-doped filter media, particularly AgCt, can provide effective protection against airborne pathogens with a lower pressure drop, elevated collection efficiency, and better disinfection capability as compared to untreated cotton filters, which are all important features for practical biocidal applications.

**Figure:**
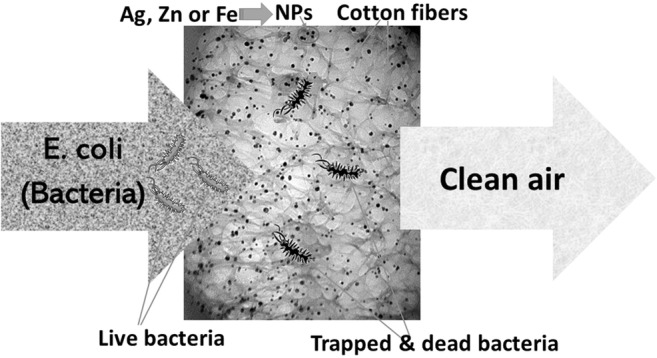
Graphical abstract

Graphical abstract

## Introduction

Exposure to particulate matter (PM) is a major concern to human health due to the potential for respiratory and cardiovascular health effects, sometimes leading to premature mortality. Particles smaller than 2.5 μm are generally considered more detrimental to human health due to their penetration capacity into the human respiratory system and ability to deposit in the bronchi and lungs (Lippmann 2012). In 2015, the World Health Assembly emphasized the necessity for the treatment of air pollution, attributing it to a cause of global health calamities. Pure and uncontaminated air is an indispensable resource for humans and is known to be crucial for safety and welfare. Growing industries and population in urban regions are rapidly worsening the air quality (Aranda et al. 2018; Rosa et al. 2017). Hence, it is the imperative that innovative techniques are developed to safeguard existing air resources and to mitigate future disasters.

Aerosols play an imperative role in spreading airborne respiratory diseases. Airborne microbes or bioaerosols (i.e. viruses, bacteria, fungi, and all biological fragments) flow freely with the airflow and can spread over a wide area in a short period of time, leading them to be a major contributor for affecting indoor air quality (Madureira et al. 2018; Lippmann 2012). Poor air quality affects productivity and downtime, contributing to health symptoms including allergies, influenza, and even sick building syndrome. Additionally, bioterrorism attacks like anthrax and the risks of pandemic airborne diseases like swine flu and avian flu have raised concerns about the health effects and airborne transport of microbes (Lee et al. 2009). Seeking a convenient, inexpensive, and efficient method for microbial decontamination is a major goal of public health against airborne biological threats. However, elimination of microbes and their infectious intensity has not been easily accomplished (Rosa et al. 2017). For this purpose, efficient, environmentally friendly and long lifetime filters are being developed by various methods in the laboratory and industrially.

An emerging idea to replace conventional filter materials for the decontamination of indoor air is the use of nanocomposite-coated biopolymers. It is well known that the properties of nanocomposite filter materials depend not only on the properties of the metal dopant but also on the morphological and interfacial characteristics from the combination with polymer material. Therefore, the use of polymers such as cellulose, starch, alginate, dextran, carrageenan, and chitosan among others have gained great importance not only due to their renewable nature and biodegradability but also because their formulations can be exploited depending on the envisaged functionality. Moreover, the application of nanocomposites (prepared simply by incorporation of functionalized nanoparticles (NPs)) onto such fibrous polymeric materials for air and water filtration are rapidly growing for environmental remediation (Yoon et al. 2008). Numerous researchers have reported the biocidal effects of various nanomaterials (Yoon et al. 2008; Lv et al. 2009;Rai et al. 2009; Rosa et al. 2017), such as silver, iron, iron oxide, and zinc oxide for their antibacterial activity (Ali et al. 2016; Aderibigbe 2017; Ali et al. 2017).

The current work illustrates the application of three different types of nanocomposite filters developed in our previous studies (Ali et al. 2017; Ali et al. 2018a) for bioaerosol control. The filters were composed of commercially available simple cotton (balls or pads which are commonly used for medical or cosmetic purposes) impregnated with silver, zinc, or iron NPs (AgCt, ZnCt, or FeCt). The decontamination performance of these filters was evaluated using aerosolized *E. coli* in a lab-scale air filtration system. Their physical removal efficiency (PRE), viable removal efficiency (VRE), and relative survival fraction under different relative humidity (RH) values were investigated. This work seeks to aid in the development and optimization of safe filters that can be applied to building air handling systems to improve indoor air quality.

## Methodology

### Test microorganism

Commonly found as normal microflora in soils, water, and living organisms, the Gram-negative bacteria *Escherichia coli* (*E. coli*) was employed as the testing microbe for this study. Gram-negative bacteria are sensitive to air flow exposure; thus, survival from aerosolization was characterized before filter tests (Lee et al. 2008). *E. coli* strain K-12 (cat. no. 15597) from the American Type Culture Collection (ATCC, Manassas, VA) was used, which is a rod-shaped bacterium, around 0.5–1.5 μm in width and 2–6 μm in length (Choi et al. 2015). First, the cultivation was carried out by inoculating a loopful of lyophilized *E. coli* onto Difco Nutrient Agar (Sigma-Aldrich, Inc., St. Louis, MO, USA), then kept for overnight incubation at 37 °C. The grown bacteria from the plate were subsequently streaked for isolation. Then, by inoculating a loopful of bacteria from an isolated colony into 50 mL of sterile Difco Nutrient Broth (NB, Sigma-Aldrich, Inc., St. Louis, MO, USA), the *E. coli* suspension was prepared and the flask was incubated at 37 °C overnight prior to use (Shiloach and Fass 2005). Prior to nebulization, 5 mL of *E. coli* stock suspension was aseptically transferred into 45 mL sterile phosphate-buffered saline (PBS, no Mg^2+^ or Ca^2+^), and vortexed for 20 s.

### Synthesis of nanocomposite impregnated cotton

**Fig. 1:**
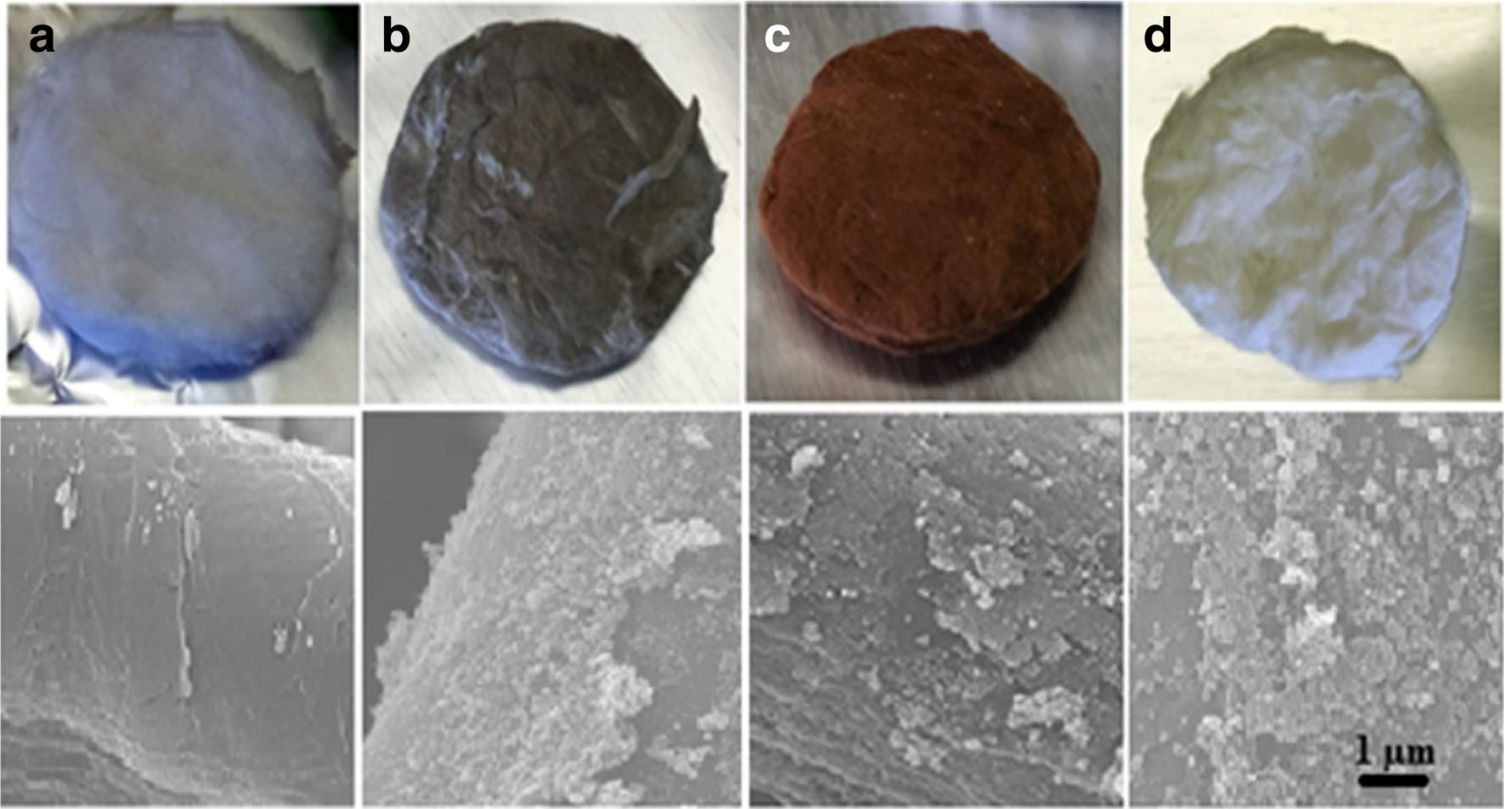
Pictorial representation of the reshaped nanocomposites in the form of filters. **a** Cotton as a control filter. **b** AgCt (cotton impregnated Silver NPs). **c** FeCt (cotton impregnated Iron-oxide NPs). **d** ZnCt (cotton-impregnated zinc-oxide NPs). Respective SEM Images at 10,000× are presented below each sample

The three nanocomposite impregnated cottons employed in this research were fabricated by following the modified protocols depicted in our previous studies (Ali et al. 2017; Ali et al. 2018a). The fibrous and fluffy nanocomposite impregnated cottons were shaped as flat filters by a facile power-driven method. Previously weighed and round-cut sample material fitting the sample holder was inserted in between two leveled, smooth 50-kg steel blocks for overnight. The samples thus obtained as nearly uniform filters (shown in Fig. 1) were then placed inside the sample holder.

### Samplers for aerosol collection

The viable efficiencies of the nanocomposite impregnated filters were examined by sampling aerosolized *E. coli* using the BioSampler (SKC Inc., Eighty-Four, PA, USA). An upgraded liquid impinger over AGI-30, the BioSampler collects microbes by inducing airborne particles to hit the swirling and churning liquid collection medium. Widely used as a reference sampler for bioaerosol sampling, its performance characteristics have been well established (Willeke et al. 1998). Its physical collection efficiency is close to 90% when sampling particles of 0.5 μm and larger (SKC, Inc.). The flow rate was maintained at12.5 l per minute (LPM), the optimal value reported in prior study (Willeke et al. 1998). Twenty milliliters sterilized DI water was added to the collection vessel, and the flow rate for each sampler was pre- and post-calibrated after each test run using a bubble-flow-calibrator (Gilian Gilibrator, Sensidyne Inc., St. Petersburg, FL).

PBS was used as the collection media for *E. coli.* Recommended by SKC Inc. for bioaerosol collection, it is non-nutritive and therefore replication is inhibited for accurate counts, and it reduces cell death by maintaining isotonic conditions. The PBS solution was prepared by diluting 10× PBS stock solution (Fisher Scientific) to 1× using sterile ultrapure deionized (DI) water (Barnstead Nanopure Diamond, 18.2 MΩ-cm). The prepared liquid was autoclaved at 120 °C for 20 min at a pressure of 15 psi. Twenty milliliters of collection liquid was used for the BioSampler and 30 mL for each testing filter material and extraction solution for *E. coli*.

A polypropylene in-line holder (cat. no. 225-1712) having instant fitting possessor for cotton material was used to house the test filter with proper openings for connection tubing. The filter sample material was compacted between two thin and round rubber gasket rings (with 2.5 cm aperture diameter) to tightly seal the holder to maintain pressure drop.

### Experimental system

**Fig. 2:**
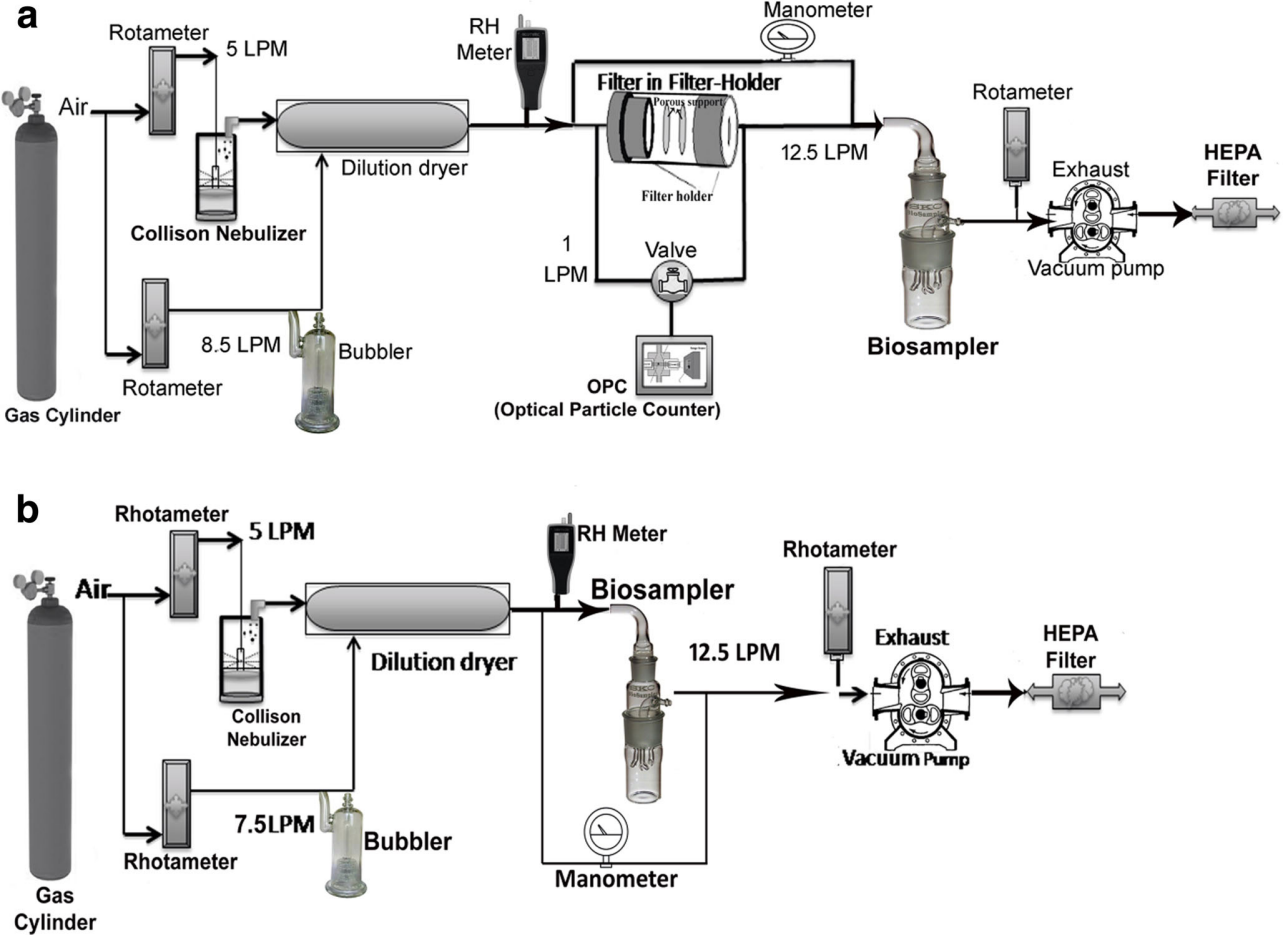
Schematic diagram of the bioaerosol testing system

Figure 2 provides a schematic diagram for the bioaerosol testing system. The *E. coli* aerosol was generated by a six-jet Collison nebulizer (Model CN25, BGI Inc., Waltham, MA) operated at 5 LPM; the lower flow rate was selected to prevent froth formation. The *E. coli* aerosol was diluted by 8.5 LPM of dry air in a dilution dryer to remove excess moisture. The resulting aerosol was then passed through the test filter material. The BioSampler downstream of the filter (Fig. 2a) captured particles that penetrated the filter. Likewise, the BioSampler was also applied singly without a filter holder to determine the maximum viable *E. coli* in the aerosol so that VRE for each filter could be assessed (Fig. 2b). Each filtration experiment was conducted for 15 min with the filter holder placed before the BioSampler (Fig. 2a). The pressure drops of the nanocomposite filters were measured using a Magnehelic gauge connected between upstream and downstream of the test filter.

A bubbler or humidifier was separately attached to the dilution dryer to increase the RH in the system. The RH inside the system was measured by an RH meter (Model HX94C, OMEGA Engineering Inc., Stamford, CT). Different RH values were evaluated (20, 30, 50, 70, and90%) to maintain an optimal RH for collecting maximum viable counts (colony forming units, CFUs).

Aerosol size distribution was obtained using an optical particle counter (OPC; Model 1.108; GRIMM Technologies Inc., Douglasville, GA). The OPC records the size distribution of particles from 0.50 to 32 μm. For these measurements, the upstream and downstream sides of filter holder were connected to the OPC by tubing, i.e., controlled and directed by a 3-way valve to record the readings for each turn of 5 min and afterward manipulated by the operating software (Control GRIMM spectrometer for aerosol). The RH was maintained at 50 ± 5% for size distribution measurement. Exhaust air was passed through a high-efficiency particulate air (HEPA) filter before discharge into a biosafety hood.

After each trial, each filter was removed from the holder and shaken for half an hour with 30 mL of PBS in a 50-mL conical tube by using a wrist action shaker (Model 75, Burrell Scientific). Afterward, 0.1 mL aliquots were taken to directly spread over Petri dishes containing Macconkey agar (Fisher Scientific) for *E. coli*. Then, the Petri dishes were incubated for 24 h at 37 °C for microbial colony counts.

Analysis of the pressure drop across each type of filter was performed using a system consisting of a manometer, a compressor, a flow meter, and a vacuum pump. All the filter materials were cut into suitable sizes for positioning on the support (diameter of 0.025 m and area of 4.91 × 10^−4^ m^2^) of the filter holder, and the flow rate across the filter medium was controlled by a rotameter calibrated by the bubble-flow-calibrator. At each flow rate, pressure drop (ΔP) was recorded for subsequent calculation of permeability, following Eq. 1 for Darcian permeability (k_1_) (Rosa et al. 2017):

1 $$ \frac{\Delta P}{L}=\frac{\mu }{k1}.V $$

where *k*_1_ is the Darcian permeability, *μ* is the viscosity of the air, *L* is the thickness of the materials measured using a Vernier caliper, and the superficial velocity (*V*) is calculated by dividing the air flow rate by the exposed surface area of the filter. The calculation used a plot of ΔP/L against the superficial velocity, where *μ*/*k*_1_ corresponded to the slope of the curve.

### Removal efficiency and survival fraction

Both PRE and VRE of the filter material were calculated according to Eq. 2 (Woo et al. 2011):

2 $$ \%\mathrm{Removal}\kern0.17em \mathrm{efficiency}=\left(1-{\mathrm{N}}_{\mathrm{E}}/{\mathrm{N}}_{\mathrm{C}}\right)\times 100 $$

where N_C_ is the concentration for the positive control part (i.e., BioSampler) and N_E_ is the concentration for the experiment part (filter materials). VRE was obtained by comparing the microbial concentrations of control (N_C_) and experimental filter (N_E_) for air filtration. For PRE, N_C_ was the number of particles entering the test filter and N_E_ was the number of particles exiting the test filter obtained by OPC.

The filter quality factor (QF), a useful criterion for comparing filters, based on the physical removal efficiency of the test filters was calculated according to Eq. 3 (Woo et al. 2011):

3 $$ \mathrm{QF}=-\mathrm{In}\;p/\varDelta P $$

where *p* is the particle penetration.

The survival fraction (SF) was defined as the ratio of the microbe in the extraction solution to the total microbe count collected on the filter (Woo et al. 2011). Relative survival fraction (RSF) was then calculated to compare the results of the nanocomposite impregnated filter with the untreated cotton control filter. Herein, the total microbes collected on the filter were determined by the nanocomposite filters of experimental count subtracted by the BioSampler count of the control flow:

4 $$ \mathrm{RSF}={\mathrm{SF}}_{\mathrm{treated}-\mathrm{filter}}/{\mathrm{SF}}_{\mathrm{untreated}-\mathrm{filter}} $$

### Dislodged NP dislodgement

This experiment was conducted to determine whether the NPs impregnated in cotton can dislodge into liquid medium or if they remain bonded strongly with the cotton material. It therefore examines stability of the impregnation to remain on the cotton material (filter). While it is less an issue in air, detachment of particles in water is easier because (1) water molecule develops stronger H-bond with other molecules and (2) removal of ions from particles in water weakens the internal configuration of particles. For this purpose, five different suspensions in separate flasks (of 100 mL) were prepared: PBS only, PBS + cotton, PBS + ZnCt, PBS + AgCt, and PBS + FeCt.0.3 g of filter materials were added to 30 mL of PBS in their respective flasks. The flasks were shaken for 1 h using the wrist action shaker. Ten milliliters of each was collected by centrifugation at 3000 rpm for 10 min in different falcon tubes. Such washes were repeated four times for each sample and completed in triplicate for all the composite materials. The water samples were vortexed and vacuum filtered (Millipore Isopore membrane filter, 0.8 μm pore diameter) to remove solid materials present in the solution for further analysis by inductively coupled plasma atomic emission spectroscopy (ICP-AES, iCAP 6200, Thermo Fisher Scientific, Waltham, MA). ICP-AES has been demonstrated to be a robust methodology for the detection and characterization of metal ion dissociation (Sportelli et al. 2017).

## Results and discussion

### Material characterization

The characterization of the AgCt, ZnCt, and FeCt filters, including Fourier-transform infrared spectroscopy, x-ray diffraction, and scanning electron microscopy, was carried out and reported in our previous studies (Ali et al. 2017; Ali et al. 2018a).

**Fig. 3:**
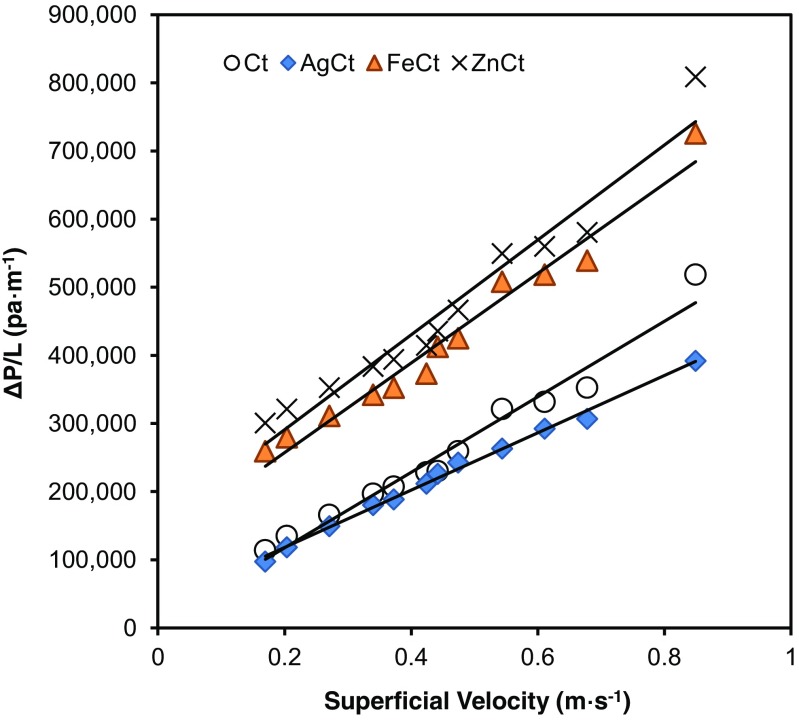
Differential pressure (Δ*P*/*L*) vs. superficial velocity for Ct, AgCt, FeCt, and ZnCt filters

As a function of superficial velocity, the Darcy’s equation was used to obtain the permeability (*k*_1_) values of the cotton and nanocomposite filter media from the plots of differential pressure (Δ*P*/*L*) vs. superficial velocity (*V*). The curves for all the treated and untreated cotton filters are shown in Fig. 3. The permeability for untreated cotton filter was measured to be 3.38 × 10^−11^ m^2^ while that for the AgCt was slightly higher as 3.64 × 10^−11^ m^2^; meanwhile, the FeCt and ZnCt filters demonstrated comparatively lower permeability as 2.06 × 10^−11^ and 1.86 × 10^−11^ m^2^, respectively. The *R*^2^ values for the cotton, AgCt, FeCt, and ZnCt filters were all greater than 0.95. Overall, the permeability values of these filters were within the range commonly found for filter media used in industrial bag filters (Flora et al. 2006; Ogulata 2006; Rosa et al. 2017).

### Air filtration

The optimal RH for collecting maximum viable counts on the filter was found to be 50 ± 5%. For *E. coli*, RH was not an important parameter for the control filter in terms of the VRE, RSF, and QF; the results under different RHs were within experimental error (Woo et al. 2011). Conversely, at medium RH (50 ± 5%), the nanocomposite filters illustrated greater VRE as compared to the results at lower RH (25 ± 5). Restraining growth and survival of microorganisms accumulated on the filter is critical in impeding re-aerosolization of collected microbes from the filter.

The pressure drops across the cotton and three nanocomposite filters are listed in Table 1. Due to the placement of downstream BioSampler, a common flow rate of 12.5 LPM was maintained for all four types of test filters by tuning the rotameters accordingly. Therefore, the optimum collection efficiencies with acceptable limit of pressure drop values were attained without penetrating the filter fabric even at a higher flow rate. Among them, the pressure drop of the AgCt filter, 254 Pa, was found to be notably lower as compared to the other two filters (ZnCt and FeCt) as well as the control filter (Table 1). Owing to negligible penetration values, the QF of each filter could not be calculated except for ZnCt as 0.005 kPa^−1^. Previous studies demonstrated a lower QF of manually fabricated filters for the bacterial elimination particularly *E. coli* as compared to the aerosols of viruses (Woo et al. 2011).

**Table 1:** Pressure drop (at Q = 12.5 LPM), physical removal efficiency (PRE), viable removal efficiency (VRE), and relative survival fraction (RSF) of control and nanocomposite filters in air filtration system

Filter	ΔP (Pa)	PRE (%)	VRE (%)	RSF
AgCt	254	97.38 ± 2.31	99.51 ± 0.23	0.0063 ± 0.0005
ZnCt	498	99.91 ± 0.07	99.12 ± 0.46	0.0153 ± 0.0054
FeCt	448	97.31 ± 2.33	98.60 ± 0.45	0.0325 ± 0.0061
Ct	274	77.42 ± 12.03	76.58 ± 3.20	0.811 ± 0.016

### Collection efficiency

**Fig. 4:**
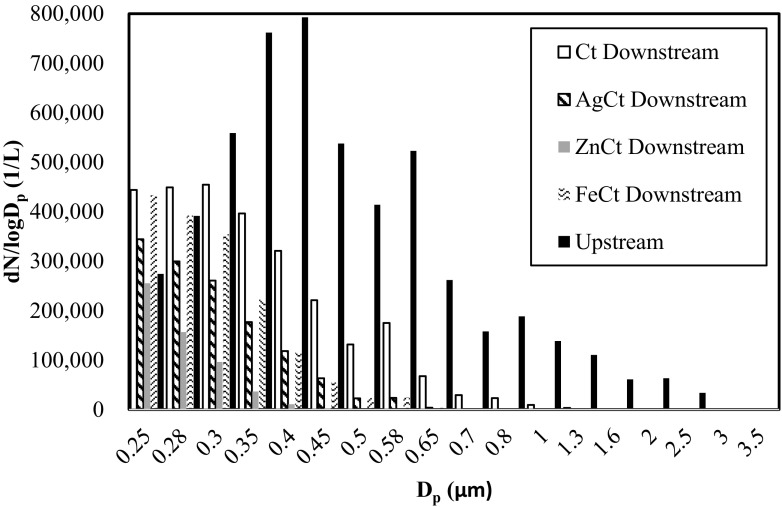
Particle size distribution of aerosols upstream of the filters (solid line) and downstream of the filters at room temperature and RH of 50 ± 5%

**Fig. 5:**
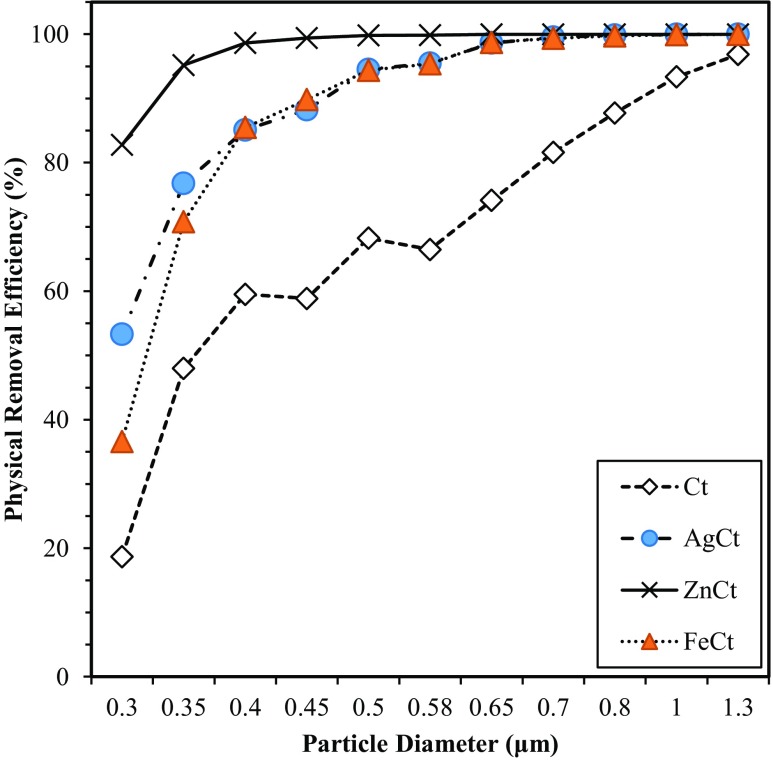
Physical removal efficiency of four different filters as a function of particle size at room temperature and RH of 50 ± 5%

The OPC provided particle size distribution in the range from 0.25 up to 32 μm, although particles larger than 6.5 μm were not detected in this study. The particle size distributions of *E. coli* aerosols upstream of the test filters had a mode at approximately 0.8 μm as shown in Fig. 4. Based on OPC measurements, ZnCt showed PRE of 100% for particles larger than 0.5 μm (Fig. 5). Overall, the average removal efficiency of three tested filters was 100% for particles larger than 0.7 μm, while the control filter barely reached 97% for particles of1.3 μm.

**Fig. 6:**
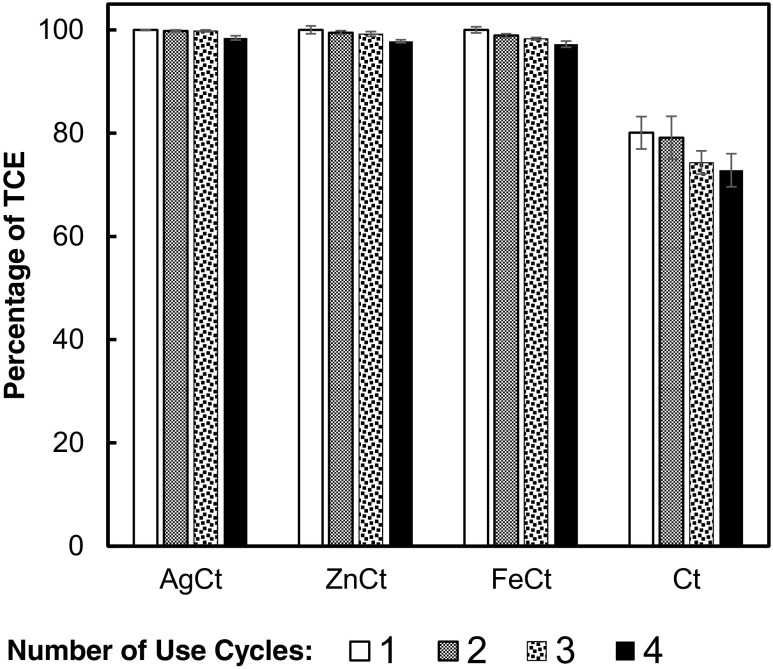
Viable removal efficiency (VRE) of *E. coli* aerosols with the nanocomposite (AgCt, FeCt, and ZnCt) and cotton (Ct) filters. The number of use cycles show the efficiency of the filter when it is recycled

VRE of the cotton and other nanocomposite filters is shown in Fig. 6. The three doped filters (AgCt, ZnCt, and FeCt) showed initial viable removal efficiency as 100% whereas the cotton control filter was only 80.09 ± 3.13%. Afterward, the nanocomposite-doped filters retained their viable efficiency (~ 99%) for four filter cycles, while the control filter demonstrated VRE of 76.6 ± 3.2%. Among the three doped filters, the AgCt illustrated the highest VRE (99.5 ± 0.23%) and the ZnCt and FeCt demonstrated 99.1 ± 0.5 and 98.6 ± 0.4%, respectively. Removal efficiencies equal to or greater than 99% are expected for industrial bag filters (Aranda et al. 2018; Rosa et al. 2017), and thus, the evaluation performance in this study supports the industrial use of the doped filters.

The difference between VRE and PRE of the control and nanocomposite filters might be attributed to the different principles of measurements and intrinsic properties of the microorganisms as well as the role of metallic NPs doped on the cellulosic surface (Scott et al. 2002). Microbial aggregates counted as one single particle by the OPC can be assayed as numerous single microbes after dispersion in the collection medium (PBS), while empty droplets counted by OPC do not result in any count by the bioassay. Additionally, environmental conditions, such as RH, may also affect the microbial viability and growth and inherently influence the difference between PRE and VRE.

### Mechanistic behavior of NPs in air filtration

NPs have been used against many disease-causing bacteria and viruses. Among the materials tested, the Ag Ct had the highest VRE and the lowest pressure drop and RSF, whereas comparatively, ZnCt performed most effectively regarding PRE. Cotton or cellulose is a suitable support material for numerous chemical groups because of the fluffy texture and sufficient availability of hydroxyl groups (Song et al. 2012). To date, only limited studies have been conducted on the activity of NP-doped cellulose (Rosa et al. 2017) or other substrates toward bioaerosols filtration (Lee et al. 2008; Jung et al. 2009; Li et al. 2011; Hamm et al. 2012; Pham and Lee 2015). The filtration efficiency of common fibrous filters enhances with the increasing filter’s solidity, which is directly proportional to the air pressure drop. Hence, with the purpose of attaining high filtration efficiency for general air filters, a greater pressure drop is unavoidable, i.e., an upshot triggering large energy losses (Fisk et al. 2002). It is recognized that the maximum effectiveness does not inevitably occur at higher airflow rates. In actuality, a high airflow velocity may increase the chance of particles penetrating the filter, rather than being captured (Liu et al. 2017). The nano-doped cotton filters tested in the present study exhibited high collection efficiencies with substantially less pressure drops, even at a high flow rate of 12.5 LPM. The PRE and VRE with their respective pressure drops are given in Table 1. The highest efficiency (99.51% VRE and 97.38% PRE) with the lowest pressure drop (254 Pa) was exhibited by the AgCt filter, while the control filter had a pressure drop of 274 Pa. This is similar to the previous studies using silver NP-coated cotton fabrics (as filters), which significantly reduced microbial growth, for the decontamination of indoor air from air conditioning systems with low pressure drop (Madureira et al. 2018; Rosa et al. 2017). In comparison, the filtration efficiency for all particle sizes has been shown to be improved with the use of many thin fibers with higher pressure drop; however, the best filter should offer the minimum pressure drop with the highest filtration efficiency (Li et al. 2016). To resolve this issue, various studies have been conducted with nanocomposite filters comprised of ultra-small fibrous material that may not create the issue of enhanced pressure drop (Liu et al. 2017).

#### Silver NPs

Silver NPs in the liquid phase were found to form pits on *E. coli* bacterial cell surfaces, and silver ions were found to remove proton motive forces between *Vibrio cholerae* bacterial cell walls (Lee et al. 2008). They have also been reported to penetrate bacterial cell walls, causing structural damage to the cell wall and cell death (Sondi and Salopek-Sondi 2004; Prabhu and Poulose 2012); produce free radicals that destroy the cell membrane, resulting in cell death (Prabhu and Poulose 2012); release silver ions which interact with the thiol groups of many vital enzymes of the bacteria, thereby inhibiting several functions in the cell (Prabhu and Poulose 2012; Lu et al. 2013). Despite increasing demand for bioaerosol control study, there have been few studies on the efficacy of silver particles in controlling bioaerosol viability. The present study manifests the utility of AgCt filter for mitigating airborne microorganisms.

#### Zinc oxide NPs

Zinc oxide NPs have been demonstrated to have good antibacterial activity against a wide range of bacteria depending upon the concentration, size, shapes, and method of preparation of the particles. Furthermore, the antibacterial activity of ZnO NPs was reported to be higher against the Gram-positive (*Staphylococcus aureus*) than the Gram-negative (*E. coli*) bacteria (Mirhosseini and Firouzabadi 2013). The difference in antibacterial activity is attributed to the particle size of the NPs, and their mechanism of antibacterial action is ascribed to damage of bacterial cell through the release of zinc (II) ions and generation of reactive oxygen species (Ali et al. 2018b; Gunalan et al. 2012). Moreover, the presence of ZnO NPs reduces the effective pore sizes of the nanocomposite filters at little expense of energy consumption. As a result, the ZnCt filter illustrated in this study excellent PRE greater than 99.9% with lower energy consumption than conventional filters (Zhong et al. 2015). Expressively, the presence of ZnO NPs strongly inhibits the propagation of bacteria on the filters. Therefore, the functionalized filters can potentially overcome the inherent limitation in the trade-off effect for controlling indoor air quality.

#### Iron oxide NPs

Although FeCt filter showed comparatively lesser antimicrobial activity as compared to AgCt and ZnCt, it did not lose its physical and viable bacterial removal efficiency after the four rinse cycles with physical (~ 98%) and viable (~ 99%) removal efficiency remaining consistent. The mechanism of the action of iron oxide NPs has also been reported by several researchers (Lee et al. 2008; Ali et al. 2016; Zheng et al. 2017). Generally, the antibacterial activity of iron oxide NPs is dependent on factors such as concentration, the type of charge on the NPs, modification of the NPs and shapes. Fe NPs are highly selective and their mode of action on bacteria is reported to be attributable to the generation of reactive oxygen species leading to lipid per oxidation, DNA damage, protein oxidation, and interaction with bacteria cell membranes via electrostatic interaction resulting in disruption of bacterial functions (Prabhu et al. 2015; Rafi et al. 2015). Importantly, the bactericidal effect of nano-iron is one of its unique properties that is not observed in other types of iron-based compounds with larger particle size (Lee et al. 2008).

### Relative survival fraction

Relative survival fraction (RSF) is an imperative aspect in exploring the reliability of nanocomposite materials as an antimicrobial filter for air filtration and understanding the mechanism. RSF was used to compare the effect of RHs on bacteria viability. Decreased bacterial count at lower RH and abundance at higher RH in control filter suggests sufficient water content enhances the endurance of aerosolized *E. coli* and thus the doped nanocomposite filters could inflict their biocidal effect on it.

The RSF values of the four filters are listed in Table 1. At 50% RH, the AgCt filter resulted in the lowest RSF of 0.0063, followed by ZnCt filter of 0.0115 and FeCt filter of 0.0325. The cotton control filter had a high RSF of 0.81. In comparison to four previously reported biocidal filters (i.e., copper, silver, TiO_2_, and iodine-treated filters) with RSF of 0.001–0.08 and 0.25–0.79 at higher RH and lower RH, respectively (Rengasamy et al. 2010). The different conditions and incubation times for bioaerosol exposure to nano-doped cotton filters make it difficult to directly make comparison of the with literature values. Additional studies using different test conditions from the current study found RSFs for iodine-treated filter and dialdehyde cellulose filters to be 0.63 and 0.43, respectively (Lee et al. 2008, 2009; Woo et al. 2011).

### Metal ions released from filters

**Fig. 7:**
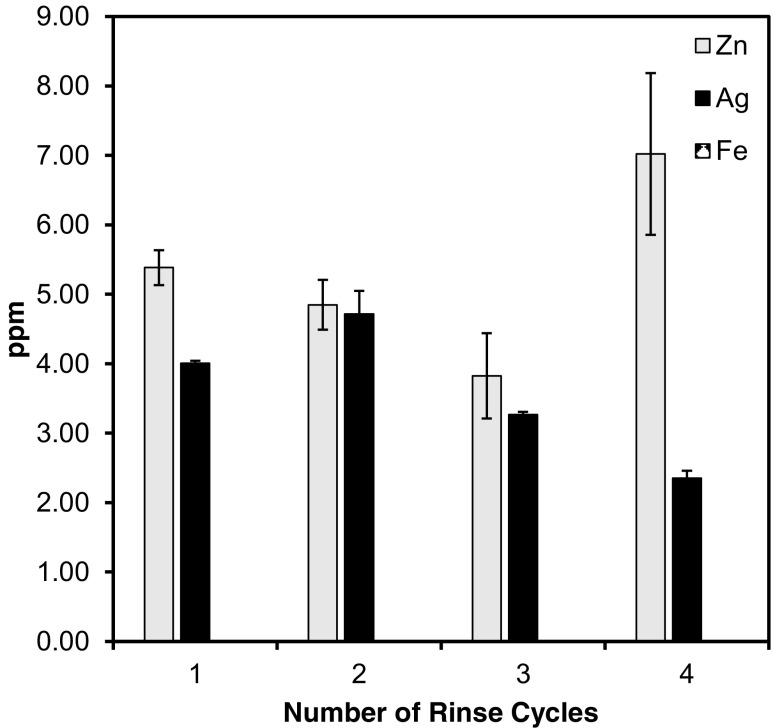
Ag, Zn, and Fe ion concentration (ppm) resulting from dissolution of metals from nanocomposite cotton filters for four reuse cycles

Four rinse-and-use cycles for the three nanocomposite filter samples were conducted to determine metal ion dissolution, and subsequent lifespan of the filters. The results are displayed in Fig. 7. The ZnCt and AgCt filters had significant ion dissolution, whereas the FeCt dissolution was below the detection limit. Generally, the metal ion dissociation decreased with rinse cycle, with an exception for zinc in its fourth rinsed cycle. Significant release of metal ions may be a major issue in the effectiveness and lifespan of nanocomposite filters for biomedical and environmental applications (Azizi et al. 2014). There was slight increase in silver ion concentration after the second rinse cycle; however, following the third and fourth rinses, it declined. Overall, the order of releasing concentration of metal ions followed Zn > Ag > Fe. It can be observed that the metal ion release was slow and was in reducing trend (Fig. 7) in each rinsed cycle despite the samples’ swelling, indicating the stable retention of the nanostructures in the fibrous matrix. Such an improved conciliation between the structural integrity in nanocomposite samples enhances their performance. Moreover, it gives a sheer indication of the durable adhesion among NPs and cellulose fibers particularly in the case of FeCt filter where iron ion has more capability to bind with cotton due to high metallic bonding where valence bonding is being involved (Zheng et al. 2017).

## Conclusions

The physical capture and biological disinfection efficiency of three different metallic NP-doped cotton biocidal filter media (AgCt, ZnCt, and FeCt) were examined as compared to cotton control filter. The results demonstrated the fabricated filter media have suitable characteristics in terms of permeability, pressure drop, and physical removal efficiency, with values similar to or better than those for filters commonly used in filtration operations. The modification of cotton with NPs was effective in inhibiting ~ 99% of the aerosolized *E. coli* for four filter cycles where they were used, rinsed, oven dried, and reused, while the control filter impeded only 76.6 ± 3.2%. A significant physical capture (> 98%) by the nanocomposite filters was observed for a wide particle size range (0.25 to 6.5 μm). There was also appreciable difference in the PRE between the NPs doped and bare cotton filters, signifying impact of the treatment.

The fibrous cotton filters doped with metallic NPs, as demonstrated, areas efficient in physical removal of aerosols as commercially available filters. None of the filters exhibited detectable penetration, even with multiple recurring experiments. No viable bacteria were observed in the shaking experiments, further supporting the efficacy of the metallic NPs to disinfect microorganisms. The high biological disinfection capacity coupled with the low pressure drops and thus high filter quality demonstrate the novel reactive filter media to be a viable alternative to conventional filtration for the removal of micron-sized bioaerosols. As the fabricated filter media were tested for four times, long-term tests to identify its effective lifetime are useful and should be explored in future studies. Furthermore, a more comprehensive testing of a variety of indoor bacteria (i.e., *Legionella pneumophila* main cause of sick building syndrome) and fungi should also be investigated.
